# Prevalence of Hypertension, Diabetes, Obesity, and High Cholesterol Among Older People Living With HIV: A Systematic Review and Meta‐Analysis

**DOI:** 10.1155/arat/6655322

**Published:** 2026-06-24

**Authors:** Mahkameh Rafiee, Soheil Mehmandoost, Fatemeh Harandi, Mohammad Sharifi, Maliheh Sadat Bazrafshani, Azam Bazrafshan, Vahid Reza Borhaninejad, Hamid Sharifi

**Affiliations:** ^1^ Student Research Committee, Kerman University of Medical Sciences, Kerman, Kerman, Iran, kmu.ac.ir; ^2^ HIV/STI Surveillance Research Center, and WHO Collaborating Center for HIV Surveillance, Institute for Futures Studies in Health, Kerman University of Medical Sciences, Kerman, Kerman, Iran, kmu.ac.ir; ^3^ Department of Medical Surgical Nursing, School of Nursing and Midwifery, Geriatric Care Research Center, Rafsanjan University of Medical Sciences, Rafsanjan, Kerman, Iran, rums.ac.ir; ^4^ Social Determinants of Health Research Center, Institute for Futures Studies in Health, Kerman University of Medical Sciences, Kerman, Kerman, Iran, kmu.ac.ir; ^5^ Institute for Global Health Sciences, University of California, San Francisco, California, USA, berkeley.edu

**Keywords:** diabetes, HIV, hyperlipidemia, hypertension, meta-analysis, obesity, older people living with HIV

## Abstract

**Introduction:**

Metabolic comorbidities are significant health challenges for older people living with HIV (PLHIV). This systematic review and meta‐analysis aimed to synthesize existing evidence and provide the pooled prevalence of metabolic comorbidities among older PLHIV worldwide.

**Methods:**

We searched PubMed, Web of Science, Embase, Scopus, and Google Scholar for English‐language documents from 2013 to 25 December 2023. To identify unpublished documents for a grey literature review, we reached out to well‐known experts in HIV and aging, asking them to provide and share unpublished records, and we searched the first 300 articles in Google Scholar. We included cross‐sectional studies and baseline data from cohort studies to measure the prevalence of metabolic comorbidities, including hypertension, obesity, diabetes, and high cholesterol. The pooled prevalence and 95% confidence interval (CI) of metabolic comorbidities were estimated using random effects meta‐analysis.

**Results:**

Of the 915 articles recruited articles for full text, 26 studies (including 10 in developed countries) met the inclusion criteria and were included in the analysis. Most of the studies were conducted in North America. The pooled prevalence of individual metabolic comorbidities was as follows: diabetes (20 articles) 13.5% (95% CI: 10.0, 17.4), high cholesterol (eight articles) 47.3% (95% CI: 36.3, 58.4), hypertension (22 articles) 40.7% (95% CI:30.2, 51.2), and obesity (10 articles) 26.1% (95% CI:20.3, 32.4). The results showed that the prevalence of all metabolic comorbidities was higher in North America.

**Conclusion:**

We found that metabolic comorbidities are a common condition among older PLHIV and represent a significant risk factor for various diseases. Policymakers should consider implementing practical interventions, including prevention strategies, periodic screenings, and lifestyle modifications focused on promoting a healthy diet and regular exercise to manage metabolic comorbidities in older PLHIV effectively. Most studies have been conducted in developed countries; there is a need for increased research efforts in other regions.

## 1. Introduction

Human immunodeficiency virus (HIV) has been associated with accelerated aging. In the literature on HIV, people living with HIV (PLHIV) aged ≥50 years are commonly defined as older PLHIV, compared to the general population’s threshold of 65 years [1, 2]. This lower cut‐off is due to the earlier onset of age‐related comorbidities and geriatric conditions in PLHIV compared to the general population [3]. According to the previous studies, older PLHIV are suffering from premature aging due to chronic inflammation and immune system activation that is happening in these people sooner than in the general population [2]. Furthermore, improvements in antiretroviral therapy (ART) have significantly reduced mortality rates and allowed these people to live longer. Despite several positive outcomes (e.g., an increase in life expectancy and an increase in quality of life), these phenomena have substantially changed the PLHIVs’ demographic, and new health‐related needs have emerged [2].

While the life expectancy of PLHIV has increased, various physical challenges have emerged [4]. Several disorders that generally affect the elderly population also appear in relatively younger PLHIV [5]. Some of these problems include motor disturbances, bone abnormalities such as osteopenia, osteoporosis, fractures [4], cardiovascular diseases (CVD), non‐HIV‐related cancers, frailty, and more frequent geriatric syndromes [6], and metabolic comorbidities [5] including hypertension, obesity, high cholesterol, and diabetes that happen in these people sooner than in the general population [7].

Metabolic comorbidities are one of the important health challenges for PLHIV. Metabolic comorbidities significantly impact health and are known as risk factors for many of the problems (e.g., hospitalization, surgery, mortality, reduced life expectancy, and increasing cardiometabolic risk factors) [7, 8]. These comorbidities are highly related to the broader health needs of older PLHIV. According to one of the previous studies, metabolic complications were observed in PLHIV after a few years of ART consumption [9]. This situation could even explain the occurrence of CVDs in this population [9]. As a result, these comorbidities can lead to a lot of unmet healthcare needs, such as the need for screening and management of related conditions, including diabetes and high cholesterol, which often are not considered in routine HIV care programs [10]. Considering the varying reports of metabolic comorbidities in recent research studies, this systematic review and meta‐analysis aimed to synthesize existing evidence and provide the pooled prevalence of metabolic comorbidities among older PLHIV worldwide. Recognizing metabolic comorbidities as one of the significant problems affecting PLHIV and understanding their prevalence can provide valuable information for developing effective healthcare programs for this population.

## 2. Methods

This systematic review and meta‐analysis used the Preferred Reporting Items for Systematic Reviews and Meta‐Analyses (PRISMA) guidelines. Prospero ID is CRD42025637380.

### 2.1. Search Strategy

We searched the following databases from 2013 to 25 December 2023: PubMed, Web of Science, Embase, Scopus, and Google Scholar for the English language documents. Appropriate Boolean operators combined three search terms related to the main topics of interest, adjusted for each database. We used MeSH terms to find the related terms. These main terms include aging (including older OR elder OR geriatric OR gerontology), HIV (including HIV OR AIDS virus OR acquired immunodeficiency syndrome OR acquired immune deficiency syndrome), and needs (includes health services need OR health need). We also conducted a manual search by reviewing the references of related articles and their citations in Google Scholar, and observed possible related articles. The complete search strategy can be found in the appendix (Supporting Information 1). We also conducted a grey literature review to identify unpublished documents and missing data. To do this, we reached out to well‐known experts in HIV and aging, asking them to provide and share unpublished records, and we searched Google and Google Scholar (for the first 300 articles) [11]. We emailed the corresponding authors, requesting them to send us the full text for unavailable articles.

### 2.2. Inclusion Criteria

We included quantitative, cross‐sectional, and baseline data in the cohort studies with original data that measured the prevalence of metabolic comorbidities, including hypertension, obesity, diabetes, and high cholesterol. The following eligibility criteria guided the study selection [1]: published in English [2], enrolled PLHIV who are older than 50 years old [3], provided a clear definition for the study population, and [4] provided estimates for the prevalence of metabolic comorbidities. For this review, older PLHIV were defined as individuals aged 50 years or older. This cut‐off aligns with the most commonly used definition in HIV research and reflects the fact that HIV‐related comorbidities and aging‐related conditions often emerge earlier in this population due to chronic inflammation and long‐term ART [12]. However, to ensure comprehensive coverage of the available evidence, we also included studies that used a cut‐off age of ≥ 60 years, as these populations fall within the broader scope of older adults and met our predefined inclusion criteria. This approach aligns with the variability in age cut‐offs reported in the literature and ensures that our review captures the full spectrum of evidence on HIV among older populations. Cohort studies that reported the prevalence of metabolic comorbidities at baseline were included.

### 2.3. Study Selection

After removing duplicates, co‐authors (M.R., S.M., and M.SH.) independently screened titles and abstracts to remove irrelevant studies. To confirm eligibility, co‐authors (M.R., F.H., and M.B.) examined studies passing the title and abstract screening process during the full‐text screening phase. Conflicts were resolved by two co‐authors (V.B. and A.B.). If disputes still existed, the senior author (H.SH.) resolved them. In the final phase, the full texts of the articles were assessed again by co‐authors (M.R., S.M., and F.H.) to determine their originality and to extract data. Their results were rechecked by other co‐authors (M.R. and M.SH.).

### 2.4. Data Extraction

Data extraction was completed independently by the first five authors. After checking the data, the authors checked their data extraction process with the senior author (H.SH) for accuracy and clarification when needed. Extracted data from the eligible full texts were input into a standardized data extraction spreadsheet. These data include [1] study characteristics (including authors of the study, year of study published, the country that the study was conducted in, sampling method, and study design) [2], participant characteristics (including study population definition, age, and sex [male vs. female]), and [3] prevalence of metabolic comorbidities. Some studies include both PLHIV and those without HIV, but we only included data on PLHIV. In contrast, other studies focused solely on PLHIV, but we again limited our inclusion to data concerning older PLHIV.

The prevalence of metabolic comorbidities was defined as the number of PLHIV who were 50 years old or older and had one of the following conditions: diabetes, high cholesterol, hypertension, or obesity. Diabetes was defined as the production of more glucose and the use of less of it, and hyperglycemia is a characteristic of the disease [13]. A diabetic person is a person with fasting plasma glucose of more than 126 mg/dL (≥ 7.0 mmol/L), random plasma glucose, and an oral glucose tolerance test of more than ≥ 200 mg/dL (≥ 11.1 mmol/L) [14]. Hypertension was defined as a blood pressure of 140/90 mmHg or higher [15]. Obesity was pragmatically defined as a body mass index (BMI) > 30 kg/m^2^. While this is a widely used and standardized metric, it does not capture central adiposity or body fat distribution, which may be particularly relevant in aging populations and PLHIV [16]. Instead of abdominal obesity, due to the lack of data about central adiposity, some of the previous research mentioned BMI as a dominant risk factor for metabolic comorbidities [17, 18]. These studies even mentioned obesity as a risk factor for hypertension, high cholesterol, and diabetes [19, 20]. High cholesterol was defined as an elevation of fasting total cholesterol concentration [21]. Recognition of high cholesterol comes from a total cholesterol of more than 200 mg/dL [22]. In some of the studies, comorbidities were reported based on medical records or diagnostic codes, reflecting real‐world clinical practice.

### 2.5. Quality Assessment

The quality of the included studies (cross‐sectional and cohort studies’ baseline) was assessed using the Newcastle–Ottawa Scale tool (adapted for cross‐sectional studies). This checklist is a tool designed to assess potential biases in various aspects of a cross‐sectional study, including study design, conduct, and data analysis [23]. The tool evaluates the following components [1]: selection of participants (maximum score of 5, assessing factors such as representativeness, sampling methods, response rate, and the use of screening tools) [2]; comparability (maximum score of 2, assessing potential confounders) [3]; and outcome (maximum score of 3, focusing on the assessment of outcomes and the statistical tests used). The total score ranges from 0 to 10, with scores categorized as follows: scores ≤ 4 are considered unsatisfactory; scores between 5 and 6 are considered satisfactory; scores ranging from 7 to 8 imply good quality; and scores of 9 or more are considered very good [24].

### 2.6. Statistical Analysis

To estimate the pooled prevalence of metabolic comorbidities, we utilized the Freeman‐Tukey double arcsine transformation to stabilize variances when generating 95% CIs (Freeman and Tukey, 1950). Given the high expected heterogeneity among prevalence studies, we opted for a random‐effects model. Consequently, we applied DerSimonian and Laird’s method (1986) for the random‐effects meta‐analysis to combine the collected data and calculate between‐study variability based on the inverse‐variance fixed‐effect model. We also conducted a meta‐regression using related factors (such as study time and study quality core) that could affect heterogeneity. The Stata meta‐prop package was used to conduct these analyses. All analyses were performed by Stata 17.0 (Stata Corp, College Station, Texas, USA) [25].

## 3. Results

### 3.1. Participants and Study Characteristics

**FIGURE 1:**
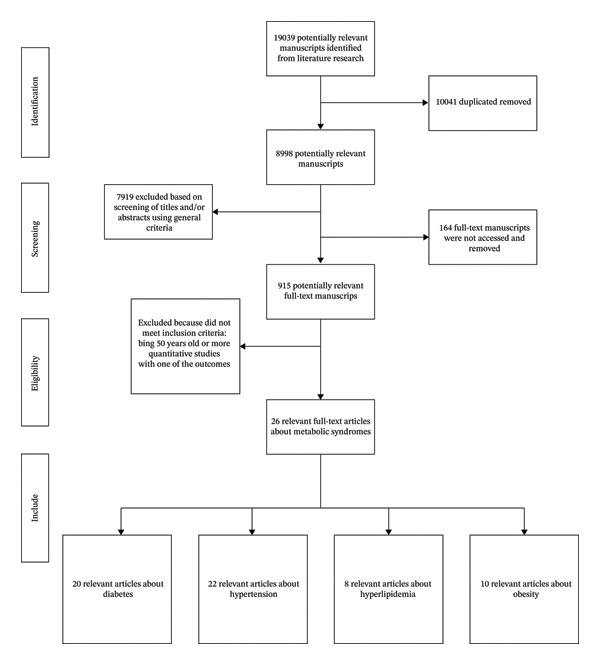
PRISMA flow diagram for selection of studies on metabolic comorbidities among older PLHIV.

In the primary search, 19,039 articles were found. After removing duplicates, 8998 studies were left from electronic databases, and one additional unpublished report through other sources was screened at the title/abstract stage; by removing unrelated articles, 915 articles were potentially relevant, and at the end, after checking the full text, 26 studies met our inclusion criteria about metabolic comorbidities. They were included in the analysis (Figure 1). Among the included studies, metabolic comorbidities are one of the problems that need to be addressed. Twenty reported the prevalence of diabetes, 22 reported the prevalence of hypertension, 10 reported the prevalence of obesity, and eight reported the prevalence of high cholesterol (Figure 1). A description of the key characteristics of included studies is provided in Table 1. The sample size of included studies ranged from 28 [26] to 19,566 [27]. A total of 20 studies were cross‐sectional, and six studies were cohort studies. Twelve of the included studies recruited participants through a convenience sampling approach; eight used medical records and census, and only one was random [28]. Studies were conducted from 2013 to 2022. Of these 26 studies, most were in North America, potentially skewing global prevalence results and limiting generalizability to the broader population of older PLHIV. One study was in Asia, one was in Australia, five were in Europe, eight were in Africa, one in South America, and ten were in North America. The results showed that the prevalence of all metabolic comorbidities is higher in North America (Table 1).

**TABLE 1:** Characteristics of included studies to estimate the prevalence of metabolic comorbidities among older PLHIV.

Authors	Country	Type of study	Population	Sample size	Method of sampling	Mean of age (SD^1^)	% men	Outcome
Mapstone, et al. (29)	US	Cross‐sectional	PLHIV^2^ from a hospital‐based infectious disease clinic	37	Convenience	58.9 (5.6)	81.8	Hypertension/ Diabetes/ High cholesterol
Greene, et al. (30)	US	Cross‐sectional	Older PLHIV who had an undetectable viral load on ART^3^	155	Convenience	NA	93.6	High cholesterol
Mugisha et al. (31)	Uganda	Cross‐sectional	Older PLHIV	244	Convenience	NA	38.8	Hypertension/ Diabetes
Solomon et al. (26)	US	Cohort	PLHIV in the late HAART^4^ era	28	Convenience	56.1 (8.9)	82	Hypertension/ Diabetes/ High cholesterol
Moore et al. (32)	US	Cross‐sectional	Older PLHIV	99	Convenience	58.7 (6.8)	87.9	Hypertension/ Diabetes/ High cholesterol
Sheppard et al (33)	US	Cross‐sectional	Older PLHIV	40	NA	54.9 (3.9)	67.5	Hypertension/ Diabetes/ High cholesterol
Underwood et al. (34)	UK	Cohort	Older PLHIV	290	Convenience	NA	88.3	Hypertension
Guaraldi et al. (35)	Italy	Cross‐sectional	Older PLHIV	1258	Convenience	72 (4.3)	83.7	Hypertension/ Diabetes
Brennan et al. (36)	South Africa	Cohort	Older PLHIV	8798	Census	NA	39.2	Hypertension
Roguet et al. (37)	France	Cross‐sectional	HIV, HBV‐ or HCV‐infected adults	46	Convenience	51.2	67.9	Obesity
Allavena et al. (38)	France	Cross‐sectional	older PLHIV	17008	Census		100	Hypertension/ Diabetes/ High cholesterol/ Obesity
Puhr et al. (39)	Australia	Cross‐sectional	PLHIV ≥ 55 years	225	NA	62.1	NA	Diabetes
Martinez‐Iglesias et al. (40)	Colombia	Cross‐sectional	Older PLHIV	669	Census	NA	71.1	Hypertension/ Diabetes/ Obesity
Obimakinde et al. (41)	Nigeria	Cross‐sectional	Older PLHIV ≥ 60 years old	62	NA	63.9 (4)	37.1	Hypertension/ Diabetes
Farahat et al. (42)	Saudi Arabia	Cross‐sectional	HIV cases at King Abdulaziz Medical City‐Jeddah	130	Census	50.1	12.6	Obesity
Rabe et al. (43)	South Africa	Cross‐sectional	PLHIV on ART > 60 years	191	Census	NA	41.9	Hypertension/ Diabetes
Justice et al (44)	US	Cohort	PLHIV from the US Veterans Affairs Healthcare System	9186	NA	NA	97.9	Hypertension/ Diabetes/ Obesity
Dakum et al. (2021) (27)	Nigeria	Cross‐sectional	Older PLHIV, on ART	19,566	Census	57.1 (6.6)	47.0	Obesity/ Hypertension/
M. M. Murray et al. (2021) (45)	US	Cross‐sectional	Older PLHIV, on ART	621	Convenience	NA	83.9	Diabetes/Obesity
Pyarali et al. (46)	US	Cohort	PLHIV	985	Census	52.2 (11.7)	55	Hypertension/ Diabetes/ High cholesterol/ Obesity
Rubtsova et al. (47)	US	Cross‐sectional	Women living with HIV	356	NA	56.5 (5.2)	0	Hypertension/ Diabetes/ Obesity
Memiah et al. (48)	Tanzania	Cross‐sectional	PLHIV	261	Convenience	NA	24%	Hypertension
Roomaney et al. (49)	South Africa	Cross‐sectional	Older PLHIV	688	Random	56	26.6	Hypertension/ Diabetes
Sharma et al. (50)	US	Cohort	Cisgender women living with HIV	164	Convenience	50 (0.5)	0	Diabetes / Obesity
Okyere et al. (51)	South Africa	Cross‐sectional	PLHIV	518	Convenience	NA	22.8	Hypertension/ Diabetes
Zanella et al. (52)	Italy	Cross‐sectional	All patients attending their HIV visit	60	Census	69.5 (13.8)	75	Hypertension/ Diabetes

^1^Standard division.

^2^People living with HIV.

^3^Antiretroviral therapy.

^4^Highly active antiretroviral therapy.

### 3.2. Quality Assessment

Most studies suffered from several methodological limitations, particularly using convenience sampling. Of the 26 included studies, 7 scored ≤ 4, indicating unsatisfactory; 12 scored 4–6, indicating satisfactory; five scored 7 to 8, indicating good quality; and two scored nine, indicating very good quality (Supporting Information 2).

### 3.3. Sensitivity Analysis by Study Quality

After quality assessment, we excluded studies rated as “unsatisfactory” on the Newcastle–Ottawa Scale and re‐ran the meta‐analyses. Excluding these studies produced minimal change in pooled prevalence estimates: hypertension decreased from 40.7% (30.2, 51.7) to 37.3% (26.1, 49.3), diabetes from 13.5% (10.0, 17.4) to 10.3% (7.2, 13.8), obesity from 26.1% (20.3, 32.4) to 27.0% (20.8, 33.6), and high cholesterol changed from 47.3% (36.2, 58.4) to 57.8% (56.8, 58.3). These differences are not significant for obesity, hypertension, and diabetes and do not change the overall interpretation; confidence intervals overlapped between original and restricted analyses, and no results reached a different conclusion. In addition, we have to mention that the increase in high cholesterol appears driven by one remaining study with a high estimate. Furthermore, we conducted a meta‐regression using study quality scores as a moderator to assess whether methodological quality influenced the pooled prevalence. The analysis revealed that study quality was not a statistically significant predictor of the heterogeneity among the studies reporting the prevalence of hypertension (*p* = 0.937), diabetes (*p* = 0.515), high cholesterol (*p* = 0.399), and obesity (*p* = 0.473); therefore, it did not improve model fit.

We conducted meta‐regression with other probable variables that could affect heterogeneity. According to the results of using study time in each study as a moderator and hypertension as an outcome in the meta‐regression, the analysis did not reveal a statistically significant association between study time and effect size (*p* = 0.206). These findings suggest that the effect size has remained relatively stable over time. We conducted a meta‐regression using the proportion of male participants in each study as the independent ariable and hypertension as the outcome, and the analysis did not reveal a statistically significant association between male proportion and effect size (*p* = 0.657). These findings suggest that sex composition among studies did not meaningfully influence the reported prevalence across studies.

We did the same for other outcomes, too. For diabetes, using study time in each study as the independent variable, the analysis reveal a statistically significant association between study time and the reported prevalence over time (DerSimonian–Laird model: β = 0.021, 95% CI: 0.010 to 0.032, p < 0.001), indicating an increase in the reported prevalence of diabetes over time. These findings suggest a potential upward trend in diabetes prevalence across studies. These findings suggest that the effect size has changed over time. In the meta‐regression using the proportion of male participants in each study and diabetes, the result did not reveal a statistically significant association between male proportion and effect size (*p* = 0.934). These findings suggest that sex composition among studies did not meaningfully influence the reported prevalence across studies.

We conducted a meta‐regression using study time in each study as the independent variable and obesity as the outcome variable. The analysis did not reveal a statistically significant association between study time and effect size (*p* = 0.805). These findings suggest that the effect size has remained relatively stable over time. The meta‐regression using the proportion of male participants in each study and obesity, the result did not reveal a statistically significant association between male proportion and effect size (*p* = 0.145). These findings suggest that sex composition did not meaningfully influence the reported prevalence across studies.

Finally, for the high cholesterol as an outcome, we conducted a meta‐regression using study time as the independent variable. According to the meta‐regression results, using study time in each study as a moderator and high cholesterol as the outcome, the analysis did not reveal a statistically significant association between study time and effect size (*p* = 0.156). We conducted a meta‐regression using the proportion of male participants in each study. The analysis did not reveal a statistically significant association between male proportion and effect size (*p* = 0.069). These findings suggest that sex composition did not meaningfully influence the reported prevalence across studies.

### 3.4. Prevalence of Hypertension

**FIGURE 2:**
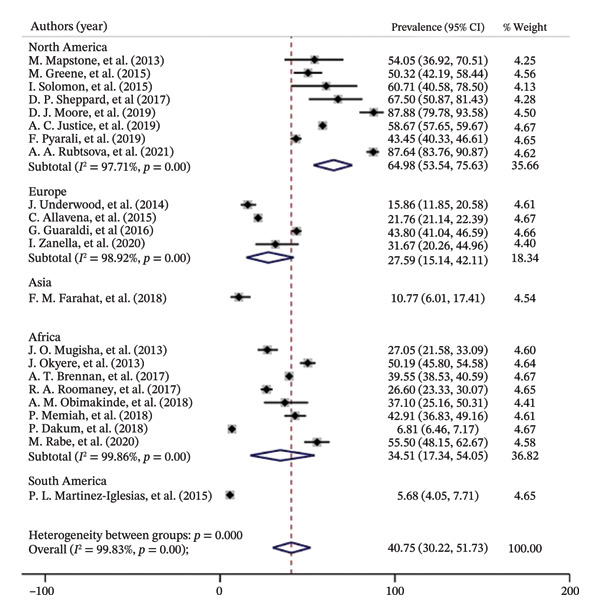
Hypertension among older PLHIV.

The prevalence of hypertension was reported in 22 articles. The prevalence of hypertension ranged from 5.7% in a study in Colombia [40] to 87.9% in a study in the US [32]. The pooled prevalence of hypertension was 40.7% (95% CI: 30.2, 51.7), and based on the geographical regions, the pooled prevalence of hypertension was higher in North America (65.5%) (Figure 2).

### 3.5. Prevalence of Diabetes

**FIGURE 3:**
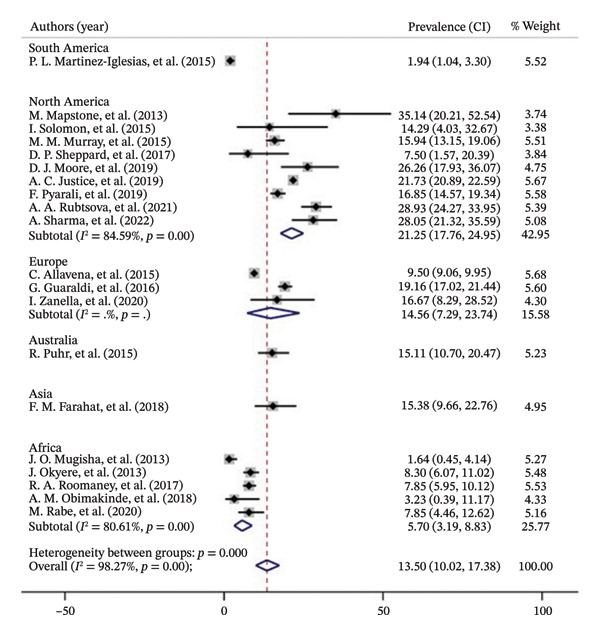
Diabetes among older PLHIV.

The prevalence of diabetes was reported in 20 articles. The prevalence of diabetes ranged from 1.9% in a study in Germany [40] to 35.1% in a study in the US [53]. The pooled prevalence of diabetes was 13.5% (95% CI: 10.0, 17.4), and based on the geographical regions, the pooled prevalence of diabetes was higher in North America (21.2%) (Figure 3).

### 3.6. Prevalence of Obesity

**FIGURE 4:**
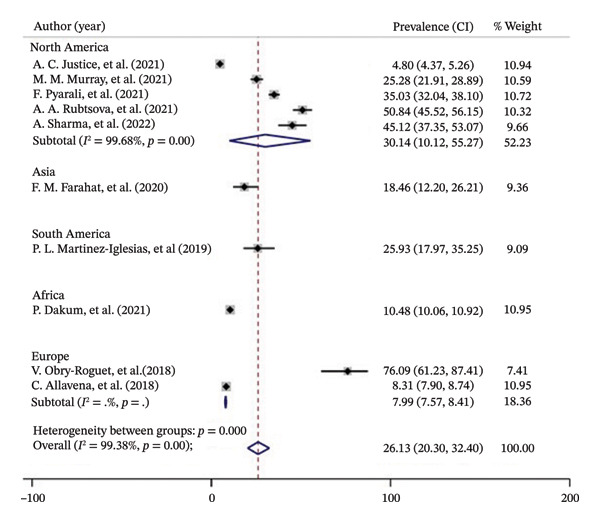
Obesity among older PLHIV.

The prevalence of obesity was reported in 10 articles. The prevalence of obesity ranged from 4.8% [44] in the US to 76.1% [37] in France. The pooled prevalence of obesity was 26.1% (95% CI:20.3, 32.4), and based on the geographical regions, the pooled prevalence of obesity was higher in North America (30.1%) (Figure 4).

### 3.7. Prevalence of High Cholesterol

**FIGURE 5:**
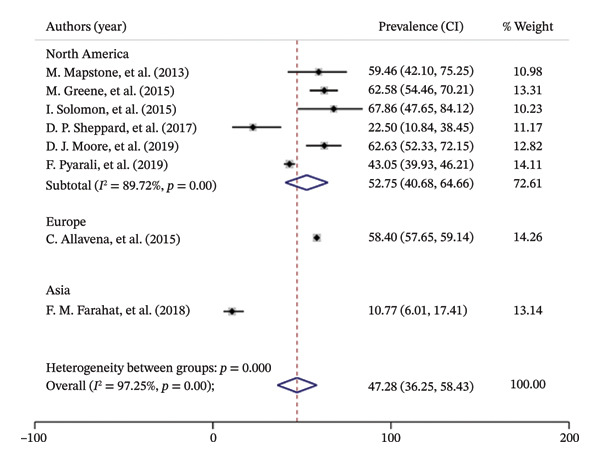
High cholesterol among older PLHIV.

Eight articles reported the prevalence of high cholesterol, which ranged from 10.8% in Saudi Arabia [42] to 67.9% in the US [26]. The pooled prevalence of high cholesterol was 47.3% (95% CI: 36.2, 58.4), and based on geographical regions, it was higher in North America (52.7%) (Figure 5).

## 4. Discussion

This systematic review and meta‐analysis estimated the pooled prevalence of metabolic comorbidities, including diabetes, hypertension, obesity, and high cholesterol. According to the results of our study, more than one in ten of the older PLHIV were diabetic, more than two in five were hypertensive, more than one in five of the participants were obese, and nearly half of the participants had high cholesterol. The highest prevalence of the comorbidities was observed in North America, where most of the studies were conducted.

As a whole, metabolic comorbidities present a highly complex and interconnected situation. Although each condition warrants individual consideration, their collective interplay must also be examined to fully understand their systemic effects because HIV and ART can lead to these comorbidities at the same time. For example, previous studies have shown that HIV could lead to hypertension through inflammation in the vascular endothelium [12]. According to a global systematic review and meta‐analysis, 25.2% of PLHIV are living with hypertension [54]. The higher prevalence of hypertension among the PLHIV who use ART is justifiable through this mechanism (36.9% vs. 23.4%) [55]. Moreover, using ART (e.g., nevirapine and efavirenz) could also increase the blood lipid profile, and lipid abnormalities were observed among those who received dolutegravir [12]. Additionally, the reversal of the catabolic state associated with uncontrolled viremia, along with the direct effects of ART, contributes to the changing rates of obesity among PLHIV [56]. Also, recurrent symptoms of peripheral fat depletion (through increasing the incidence of high cholesterol) [12] were more prevalent among the PLHIV who used zidovudine and stavudine. Furthermore, we should notice that these comorbidities can lead to each other; there is a mutual association between obesity and inflammation and deposition of ectopic lipids, which can lead to hypertension and high cholesterol [56]. Both the virus and ART (e.g., Efavirenz) could lead to metabolic dysfunction and the development of Type 2 diabetes [57, 58]. Also, insulin resistance is known as another disorder caused by HIV. The interconnection of these disorders highlights the importance of multidisciplinary approaches for treatment [12]. As a result, due to the co‐occurrence of HIV and aging comorbidities, HIV and geriatric co‐management is needed for older PLHIV, requiring expert health care providers in both HIV and geriatric fields. Additionally, comprehensive assessments, support for all of the health aspects, and targeted research to address their specific needs are required according to this co‐management.

These metabolic comorbidities are known as important risk factors for several diseases (e.g., blindness, cardiovascular, brain, renal diseases, etc.) [59]. Managing these comorbidities could prevent several health‐related problems. For example, to reduce the effect of obesity on PLHIV, a comprehensive understanding of how ART and HIV affect fat partitioning and the metabolic function of adipose tissue could be considered as an important factor in the treatment procedure for clinicians [56]. Focusing on the lifestyles of PLHIV and incorporating exercise, adopting a balanced and low‐fat diet can help reduce the risk of obesity and high cholesterol. Educating older PLHIV about the negative effects of high cholesterol can encourage them to pay more attention to medical advice and prevent its occurrence. This proactive approach can help prevent more challenging situations and adverse outcomes even after diagnosis. Additionally, comprehensive screening can help identify diabetic and prediabetic patients early, allowing for more targeted and effective interventions to manage and prevent the progression of the disease and its undeniable side effects. Noncommunicable diseases should be taken into account in the management of HIV patients to mitigate their adverse effects.

Our findings reveal a high concurrent burden of hypertension, dyslipidemia, diabetes, and obesity in older PLHIV. Clinically, these conditions rarely occur in isolation; their co‐occurrence suggests a synergistic increase in the risk for atherosclerotic CVD, accelerated vascular aging, and geriatric syndromes like frailty, beyond the sum of their individual effects [60, 61]. This interplay, potentiated by chronic HIV inflammation and ART effects, underscores that managing single conditions in isolation is an insufficient clinical strategy [12]. Consequently, public health recommendations must evolve beyond generic screening and lifestyle advice. Healthcare systems should develop and implement integrative care models tailored to this population. We advocate for HIV and geriatric co‐management programs, where care is delivered by multidisciplinary teams trained in both HIV medicine and geriatrics. Such models would employ comprehensive geriatric assessments to be integrated into routine HIV clinic visits, systematically evaluating not only metabolic parameters but also functional status, cognitive health, polypharmacy, and social determinants. Furthermore, existing chronic disease management platforms within health systems could be adapted to centrally coordinate care for older PLHIV with multimorbidity. This paradigm shifts from disease‐specific to person‐centered; integrated care is essential to improve health outcomes and quality of life for the older PLHIV.

Our study results showed that the prevalence of all metabolic comorbidities is higher in North America, which might skew the global pooled prevalence to a higher estimation. According to the results, more than half of the older PLHIV are suffering from hypertension or high cholesterol, nearly two in ten are suffering from diabetes, and more than three in ten are suffering from obesity. These results highlight several differences between the US and other countries. For example, it could be due to the higher ART coverage in North America, where 74% of the diagnosed people receive ART [62]. Previous studies have shown that ART can lead to higher metabolic comorbidities among PLHIV [12, 55, 57, 58]. We should also notice that ART use increases life expectancy, and the incidence of these comorbidities is more likely among older people [2]. Further research is required to study the global distribution of metabolic comorbidities, specifically in low‐ and middle‐income countries (LMICs). Moreover, as the ART uptake is increasing, screening programs need to be developed in these countries.

### 4.1. Limitations

We acknowledge the limitations of this study. First, inconsistent definitions of the primary outcome in the included studies, which relied on self‐report or patient charts and records, may have introduced bias and led to heterogeneity across studies. We attempted to manage these limitations by relying on clinical charts and records as a data source and by using the definitions provided by the authors. However, we could not run a sensitivity analysis comparing “lab‐defined” versus “record‐defined” comorbidities because some studies used “record‐defined” comorbidities based on existing lab results. The same problem exists about variations in ART regimens across studies that could not be assessed due to a lack of data in the included studies. Second, the predominantly male sample, sampling method including convenience sampling, and unreported mean age in several studies limit generalizability. Third, restricting inclusion to published English‐language papers may have introduced language bias and excluded relevant data, particularly from LMICs. Additionally, we have to consider the possibility of low access to data of older PLHIV in LMICs, where aging with HIV is an emerging issue. Although the high number of studies from high‐income countries such as North America may help reduce this bias. We conducted a subgroup analysis across different continents to reduce heterogeneity. Fourth, all of the studies included were cross‐sectional, and as a result, the establishment of temporal and causal relationships is not available. Finally, as noted in the quality assessment, several studies had methodological limitations. We retained these studies to preserve statistical power and avoid selection bias, but this may affect the precision of our pooled estimates.

## 5. Conclusion

Metabolic comorbidities, including hypertension, diabetes, high cholesterol, and obesity, can lead to many consequences among older PLHIV. As a whole, increasing awareness and education for PLHIV is essential. This information should include data on healthy lifestyles, such as encouraging them to engage in regular exercise (including aerobic, resistance, flexibility, and balance training), weight management, and participation in exercise programs. Additionally, it is crucial to have a monitoring system for screening risk factors to decrease the likelihood of metabolic comorbidities and regular medical check‐ups to mitigate their outcomes. This screening system could incorporate waist circumference, BMI, blood pressure, lipid profile, glucose levels, and behavioral data collected during routine healthcare visits. While most studies on this topic have been conducted in developed countries, particularly the United States, there is a need for increased research efforts in other regions. As access to ART expands globally, the number of older PLHIV is expected to rise, highlighting the urgent need for tailored interventions in these countries. We recommend further studies to investigate the impact of geographical and temporal variations on the observed associations.

## Author Contributions

Co‐authors (Mahkameh Rafiee, Soheil Mehmandoost, and Mohammad Sharifi) independently screened titles and abstracts to remove irrelevant studies. To confirm eligibility, studies passing the title and abstract screening process were examined by co‐authors (Mahkameh Rafiee, Fatemeh Harandi, and Maliheh Sadat Bazrafshani) during the full‐text screening phase. Conflicts were resolved by two co‐authors (Vahid Reza Borhaninejad and Azam Bazrafshan). If conflicts still existed, the senior author (Hamid Sharifi) resolved them. In the final phase, the full texts of the articles were assessed again by co‐authors (Mahkameh Rafiee, Soheil Mehmandoost, and Fatemeh Harandi) to determine their originality and to extract data. Their results were checked again by other co‐authors (Mahkameh Rafiee and Mohammad Sharifi).

## Funding

This study was funded by the Kerman University of Medical Science (Grant no. 402000756).

## Disclosure

All authors have read and approved the final manuscript.

## Ethics Statement

The authors have nothing to report.

## Consent

The authors have nothing to report.

## Conflicts of Interest

The authors declare no conflicts of interest.

## Supporting Information

Additional supporting information can be found online in the Supporting Information section.

## Supporting information


**Supporting Information** Appendix description for Supporting Information: Supporting Information 1: PubMed search strategy. This appendix provides the detailed search strategy that was used for PubMed. Other databases used the same keywords but with their own searching rules. Supporting Information 2: Quality Assessment of Articles Using the Newcastle–Ottawa Scale. This appendix provides the detailed quality assessment scores for all studies included in the review, evaluated using the Newcastle–Ottawa Scale. For each nonrandomized study, the Newcastle–Ottawa Scale assesses three domains: selection of study groups (0–4 stars), comparability of groups (0–2 stars), and ascertainment of exposure or outcome (0–3 stars). The total Newcastle–Ottawa Scale score (range: 0–9 stars) is reported, with studies scoring ≥ 7 considered to have high methodological quality. This assessment supports the risk‐of‐bias evaluation discussed in the main manuscript.

## Data Availability

Data sharing does not apply to this article as no new data were created or analyzed in this study.
