# Supplementary material for: Prevalence of Hypertension, Diabetes, Obesity, and High Cholesterol Among Older People Living With HIV: A Systematic Review and Meta‐Analysis
Source: AIDS Res Treat. 2026 Jun 24;2026:6655322. doi: 10.1155/arat/6655322 (PMC13292179; doi:10.1155/arat/6655322)
Supplement: Supplementary file 1 — Supporting Information Appendix description for Supporting Information: Supporting Information 1: PubMed search strategy. This appendix provides the detailed search strategy that was used for PubMed. Other databases used the same keywords but with their own searching rules. Supporting Information 2: Quality Assessment of Articles Using the Newcastle–Ottawa Scale. This appendix provides the detailed quality assessment scores for all studies included in the review, evaluated using the Newcastle–Ottawa Scale. For each nonrandomized study, the Newcastle–Ottawa Scale assesses three domains: selection of study groups (0–4 stars), comparability of groups (0–2 stars), and ascertainment of exposure or outcome (0–3 stars). The total Newcastle–Ottawa Scale score (range: 0–9 stars) is reported, with studies scoring ≥ 7 considered to have high methodological quality. This assessment supports the risk‐of‐bias evaluation discussed in the main manuscript. [file ARAT-2026-6655322-s001.docx]

**Supplementary material:**

**Supplementary material 1: PubMed search strategy**

| # | Syntax | Results |
| --- | --- | --- |
| 1 | ("older"[Title/Abstract] OR "elder*"[Title/Abstract] OR "aging"[Title/Abstract] OR "geriatric*"[Title/Abstract] OR "gerontology"[Title/Abstract]) | 1,041,100 results |
| 2 | ("HIV"[Title/Abstract] OR "human immunodeficiency virus"[Title/Abstract] OR "aids virus*"[Title/Abstract] OR "acquired immunodeficiency syndrome"[Title/Abstract] OR "acquired immune deficiency syndrome"[Title/Abstract]) | 391,557 results |
| 3 | ("need*"[Title/Abstract] OR "health services need*"[Title/Abstract] OR "health need*"[Title/Abstract]) | 2,432,706 results |
| 4 | 1 AND 2 AND 3 | 3,980 results |

**Supplementary material 2:** **Quality Assessment of Articles Using the Newcastle-Ottawa Scale.**

|  | **selection** | | | | **Comparability** | **outcome** | | **Score** | **quality** |
| --- | --- | --- | --- | --- | --- | --- | --- | --- | --- |
| **Authors** | **Q1** | **Q2** | **Q3** | **Q4** | **Q5** | **Q6** | **Q7** | **Total** |  |
| M. Mapstone, et al. (2013) | 1 | 0 | 0 | 1 | 0 | 1 | 1 | 4 | Unsatisfactory |
| M. Greene, et al. (2015) | 1 | 0 | 0 | 0 | 0 | 1 | 1 | 3 | Unsatisfactory |
| J. O. Mugisha et al. (2016) | 1 | 1 | 0 | 1 | 1 | 1 | 1 | 6 | Satisfactory |
| I. Solomon et al. (2016) | 0 | 0 | 0 | 0 | 0 | 0 | 1 | 1 | Unsatisfactory |
| D. P. Sheppard et al (2017) | 0 | 0 | 0 | 0 | 0 | 1 | 1 | 2 | Unsatisfactory |
| J. Underwood et al. (2017) | 1 | 0 | 0 | 0 | 0 | 0 | 1 | 2 | Unsatisfactory |
| D. J. Moore et al. (2017) | 1 | 0 | 0 | 0 | 1 | 1 | 1 | 4 | Unsatisfactory |
| G. Guaraldi et al (2018) | 1 | 1 | 0 | 2 | 0 | 2 | 1 | 7 | Good |
| O. Roguet, et al (2018) (40) | 1 | 0 | 0 | 1 | 0 | 2 | 1 | 5 | Satisfactory |
| C. Allavena et al. (2018) | 1 | 1 | 0 | 1 | 0 | 2 | 1 | 6 | Satisfactory |
| A. T. Brennan et al. (2018) | 1 | 1 | 0 | 2 | 0 | 2 | 1 | 7 | Good |
| A. Margaret Obimakinde et al. (2018) | 0 | 0 | 0 | 2 | 0 | 2 | 1 | 5 | Satisfactory |
| R. Puhr et al. (2019) | 1 | 0 | 0 | 1 | 0 | 1 | 1 | 4 | Unsatisfactory |
| P. L. Martinez-Iglesias et al. (2019) | 1 | 1 | 1 | 0 | 0 | 2 | 1 | 6 | Satisfactory |
| F. M. Farahat, et al. (2020) | 1 | 0 | 0 | 1 | 0 | 2 | 1 | 5 | Satisfactory |
| M. Rabe et al. (2020) | 1 | 1 | 0 | 0 | 0 | 2 | 1 | 5 | Satisfactory |
| M. M. Murray et al. (2021) | 1 | 1 | 0 | 1 | 1 | 1 | 1 | 6 | Satisfactory |
| Dakum et al. (2021) | 1 | 1 | 1 | 0 | 0 | 2 | 1 | 6 | Satisfactory |
| A. C. Justice et al. (2021) | 0 | 0 | 1 | 2 | 1 | 2 | 1 | 7 | Good |
| F. Pyarali et al. (2021) | 1 | 1 | 1 | 2 | 1 | 2 | 1 | 9 | Very good |
| A. A. Rubtsova et al. (2021) | 1 | 0 | 1 | 1 | 1 | 2 | 1 | 7 | Good |
| P. Memiah et al. (2021) | 1 | 1 | 1 | 2 | 0 | 2 | 1 | 8 | Good |
| A. Sharma et al. (2022) | 0 | 0 | 0 | 2 | 0 | 2 | 1 | 5 | Satisfactory |
| R. A. Roomaney et al. (2022) | 1 | 1 | 0 | 0 | 0 | 1 | 1 | 4 | Unsatisfactory |
| J. Okyere et al. (2022) | 1 | 1 | 1 | 1 | 0 | 1 | 1 | 6 | Satisfactory |
| I. Zanella et al. (2022) | 1 | 1 | 1 | 1 | 0 | 2 | 1 | 7 | Good |
